# Case Report: Dendritic Cells and Macrophages Capture Sperm in Chronically Inflamed Human Epididymis

**DOI:** 10.3389/fimmu.2021.629680

**Published:** 2021-02-23

**Authors:** Wenzhong Zheng, Shiqiang Zhang, Xiaobao Chen, Shaoqin Jiang, Zhihao Li, Mengqiang Li

**Affiliations:** ^1^ Department of Urology, Fujian Medical University Union Hospital, Fuzhou, China; ^2^ Department of Urology, Kidney and Urology Center, The Seventh Affiliated Hospital, Sun Yat-sen University, Shenzhen, China

**Keywords:** chronic inflammation of genital tract (CIGT), oligozoospermia and asthenospermia, chronic epididymitis, macrophages, dendritic cells

## Abstract

Chronic inflammation of the male genital tract is thought to be a primary etiological factor of male infertility. The abundance and activation of macrophages and dendritic cells in patients with chronic inflammation of genital tract were closely associated with oligozoospermia and asthenospermia. Chronic epididymitis appears to be more important than seminal vesiculitis or prostatitis due to the direct interaction between spermatozoa and epididymal inflammatory cells. In this study, we present a case report of a 41-year-old male with oligoasthenospermia and chronic epididymitis. Hematoxylin-eosin staining and immunofluorescence analyses showed that antigen presenting cells including macrophages and dendritic cells were found capturing spermatozoa in the lumen of cauda epididymis. To our knowledge, this is the first case report that directly observed dendritic cells capturing spermatozoa in the lumen of an inflamed epididymis. This finding directly explains chronic epididymitis as the possible cause of oligospermia in patients.

## Introduction

Infertility affects approximately 15% of couples, and male factor infertility account for approximately 20~50% of all infertile patients (1, 2). Teratozoospermia, azoospermia, oligozoospermia, and asthenospermia are the main causes of male infertility, accounting for 20~25% of male cases (3). Inflammation of the male genital tract is thought to be a primary etiological factor of oligozoospermia and asthenospermia (4–6). Chronic epididymitis appears to be more important than seminal vesiculitis or prostatitis due to the direct interaction between spermatozoa and epididymal inflammatory cells (7–9).

Normal epididymis environment contains an intricate network of antigen presenting cells including residual dendritic cells and macrophages (10). The identification and removal of abnormal spermatozoa or the possible presence of sperm “mass control” mechanisms is one of the most interesting field of research for epididymal physiology (11, 12). Some earlier studies have presented evidence that the obstruction of genital tract was associated with spermatophagy in human and monkey epididymis (13). Although the “spermatophagy” phenomenon has been previously reported (13), there had been no convictive evidence presented, until recently, that dendritic cells and macrophages play an important role in the identification and removal of defective sperms in normal epididymis. Recent studies showed that the abundance and activation of dendritic cells in patients with chronic inflamed epididymitis were closely associated with oligozoospermia and asthenospermia (14, 15). However, evidence of dendritic cells and macrophages participation in sperm damage in epididymitis has not been presented in chronically inflamed human epididymis.

## Case Presentation

**Figure 1:**
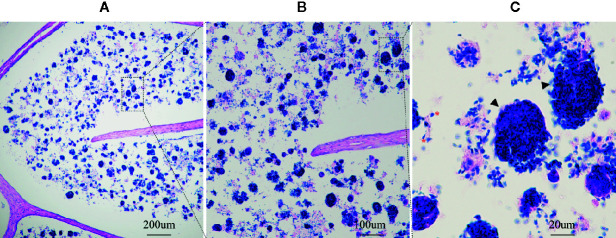
Sperm-attached cells in the lumen of inflamed epididymis. Magnification: ×10 **(A)**, ×40 **(B)**, and ×100 **(C)**; * represent the spermatozoa; Triangle represent the infiltrating cells.

**Figure 2:**
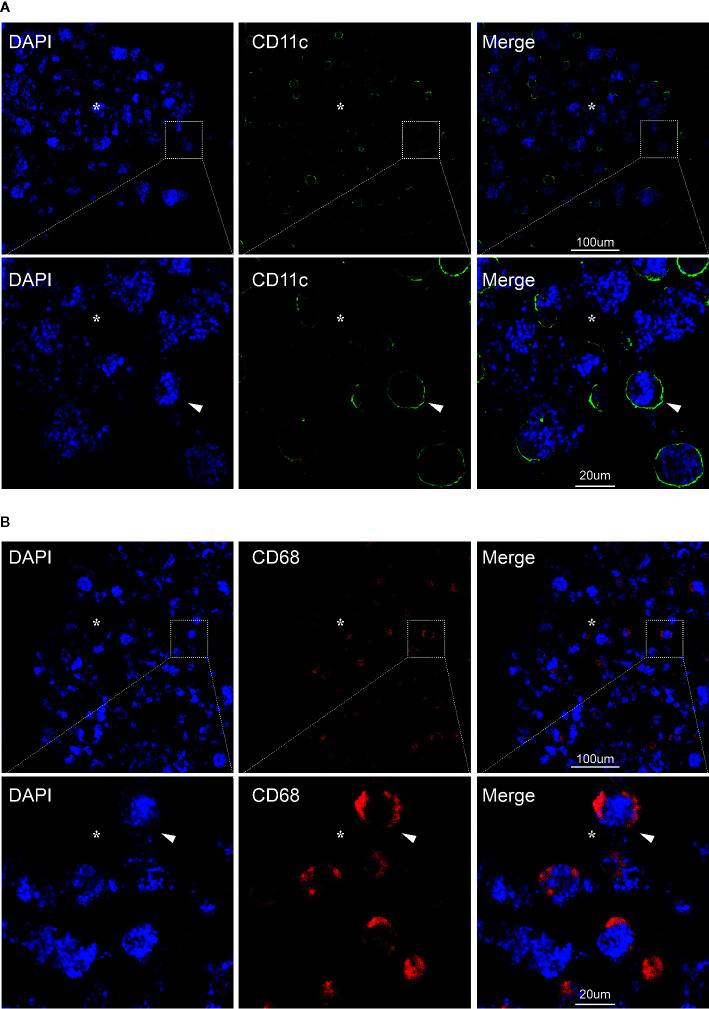
CD11c+ dendritic cells and CD68+ macrophages in the lumen of chronic inflamed epididymis. Immunofluorescence staining of CD11c+ dendritic cells **(A)** and CD68+ macrophages in the lumen of human epididymis **(B)**; Nuclei were labeled with DAPI; CY3, red fluorescence; FITC, green fluorescence; Arrows represent the positive cells; *represent the lumen of epididymis; Bar = 100 µm (or 20 um); Original magnification: ×100; further magnification: ×400.

Here we present a case report of a 41-year-old male with oligoasthenospermia and chronic epididymitis. This male patient is a driver and visited the outpatient department presenting with the complaints of bilateral testicular burning pain for 9 months. Scrotal color Doppler ultrasound showed chronic bilateral inflammatory changes of bilateral epididymis and he was primary diagnosed with chronic bilateral epididymitis. In addition, the patient was diagnosed with oligozoospermia and asthenospermia based on semen samples according to the WHO criteria (WHO laboratory manual for the examination and processing of human semen—5th edition). The parameters of semen were evaluated by a computer-aided sperm analysis (CASA) (NatureGene Corp, Sperm Tracker, USA) and was presented in **Table 1**. This man did not report any disorders with penile erections, orgasm, or ejaculation and he was not taking any medication for any systemic diseases including diabetes mellitus, hypertension, etc. Physical examination showed bilateral testes were in normal position and tender upon palpation. The rest of the physical examination was normal. The patient was referred to many hospitals for conservative treatment including general treatment, medication, physical therapy, and Traditional Chinese Medicine treatment, and the symptoms of pain was not effectively relieved and in fact worsened, which strongly affected his sleep, diet, mental state, etc. The patient already has two children and are willing to receive epididymectomy. Therefore, bilateral epididymectomy was performed at the Urology Center of our hospital on September 8, 2016. Excised epididymal tissue samples were immediately fixed in Bouin’s solution and embedded in paraffin for Hematoxylin-eosin staining and immunofluorescence analyses (16). Hematoxylin-eosin staining was carried out following standard protocols. Briefly, paraffin-embedded epididymal biopsies (4 μm sections) were dewaxed (dimethylbenzene), hydrated (100, 90, 80, 75, and 70% alcohol gradient) and stained in hematoxylin solution for 3 min. The slice was then decolorized in acid alcohol solution (0.4% HCL in EtOH) for 10 s in hydrochloric acid ethanol differentiation buffer solution, washed in pho-phate-buffered saline (PBS, pH 7.45), and stained with eosin solution for 2 min. After sealing with neutral resin seal, the slides were examined under a digital microscope (Olympus BX 53 microscope, Tokyo, Japan). Interestingly, we observed large numbers of spermatozoa-attached cells in the lumen of inflamed epididymis which indicated that those spermatozoa were directly captured and degraded by infiltrating cells during the process of chronic epididymitis (**Figures 1A–C**, **Supplementary Figure 1** **)**. In addition, we further analyzed the immune phenotype of infiltrating cells in the lumen of inflamed epididymis by performing immunofluorescence analyses. Immunofluorescence staining was carried out by the following steps. Specifically, the prepared paraffin-embedded biopsies were fully immersed in xylene (I) for 10 min, xylene (II) for 10 min, anhydrous ethanol (I) for 5 min, anhydrous ethanol (II) for 5 min, 95% alcohol for 5 min, 90% alcohol for 5 min, 80% alcohol for 5 min, 70% alcohol for 5min, and then soaked in distilled water for 1 min. Antigen retrieval was carried out with Tris-EDTA (pH 9.0) buffer solution for 30 min in a temperature control water bath. The sections were placed in 0.3% hydrogen peroxide solution for 15 min to eliminate the influence of endogenous peroxidase. Then 5% goat serum was added and the tissues were incubated at room temperature for 1 h to block non-specific sites. The primary antibody of antigen presenting cells associated markers including CD68 (66231-1-Ig, IgG1; ProteinTech, Wuhan, CHN) and CD11c (60258-1-Ig, IgG2a; ProteinTech, Wuhan, CHN) was added and then stored overnight at 4°C environment. Incubate the tissue for 1 h with the secondary antibody in the dark at room. The tissues were incubated with DAPI for 15 min. The sections were mounted with anti-fluorescence quencher, and were visualized under a confocal microscope (ZEISS LSM 880 confocal microscope, Jena, Germany). Immunofluorescence staining results showed that both the CD11c+ dendritic cells and CD68+ macrophages were found capturing spermatozoa in the lumen of the inflamed epididymis (**Figures 2A, B**). In this case report study, the negative controls were carried out by substituting the primary antibodies with its corresponding isotype control including IgG or IgG1, respectively, during the immunofluorescence staining process (data not shown).

**Table 1:** The parameters of semen.

Volume	PH	Liquefaction time	Color	Sperm concentration	WBC	Round cells	Neutral α-glucosidase
2.0 ml	7.45	50	Gray white	2.5 × 10^3^/ml	3.0 × 10^6^/ml	9.0 × 10^6^/ml	11.94 IU/per ejaculation
**Total activity**	**Fast forward motion**	**Survival rate** **(live sperm)**	**Teratogenesis rate**	**Fructose**	**Acid phosphatase**	**Zn^2+^**	**MAR test**
0%	0%	0%	100%	6.04 g/per ejaculation	396.6 U/per ejaculation	3.8 mmol/per ejaculation	5%

WBC in semen were identified by toluidine blue oxidase staining in this study. MAR, represent mixed antiglobulin reaction; WBC, represent white blood cell; a, represent fast forward movement; b, represent slow forward motion; c, represent non-forward motion; d, represent not moving sperm.

## Discussion

Current studies on male infertility mostly focuses on the spermatogenic origins, whereas researches on the role of the rest of the male genital tract remains relatively sparse. Epididymis is a male-specific organ where sperms gain the ability to fertilize an oocyte in order to development an embryo (17). In addition, both testis and epididymis are considered as peripheral immune tolerant to the immunologically foreign spermatogenic cells and spermatozoa (18). Importantly, the incidence of immune tolerance shift in the epididymis micro-environment is more prevalent than the testicular micro-environment, as the elaborate blood-epididymis barrier appears to be more vulnerable compared to the blood-testis barrier (19). Under a certain condition, the blood-epididymis barrier and the residual immune cells of human epididymis can cooperate in an intricate network to prevent immunologically antigens from entering the peripheral circulatory system and initiating a pathological immune defense against spermatozoa (20).

Immunological balance shift in the vas deferens, epididymis, and testis can have adverse effects on spermatozoa and inhibits male reproduction (21). Dysfunction of immune tolerance to sperm in human epididymis leads to the development of sperm-associated autoantibodies and is a considerable problem, resulting in oligozoospermia and asthenospermia which account for 5~10% of male infertility cases (22). Professional antigen-presenting cells including dendritic cells and macrophages are a versatile manager between innate and adaptive immunity system, which promoting initiate specific defense mechanisms by recruiting antigen-specific T lymphocytes (23). An intricate network of dendritic cells and macrophages was detected in mouse epididymis and are especially intensive in the caput region, indicating that these cells are particularly active in the caput of epididymis (24). Our previous study showed that dendritic cells with a potentially tolerogenic phenotype is especially numerous in the caput region of human epididymis (15). It is possible that these dendritic cells and macrophages in caput epididymis play a role in preventing sperm autoimmunity (25). Overall, the number and structural prominence of dendritic cells and macrophages in epididymis are much greater in the caput region than either in the cauda or corpus, and this needs to be considered in conjunction with the quantitatively different responses of the caput and corpus (or cauda) to immunological challenges such as chronic epididymitis.

An earlier study has showed that the macrophages in the obstruction genital tract was associated with spermatophagy in epididymis (13). Although the “spermatophagy” phenomenon has been previously reported, there had been no convictive evidence presented, until recently, that dendritic cells and macrophages are associate with spermatophagy in chronic epididymitis. In this case report, we directly observed large numbers of spermatozoa-attached cells in the caput region of chronic epididymitis samples which indicated that during the process of chronic epididymitis sperm might be directly captured and degraded by infiltrating immune cells. In addition, our further immunofluorescence analysis showed that both the CD11c+ dendritic cells and CD68+ macrophages were found capturing spermatozoa in the lumen of epididymis which might explain the cause of oligospermia in patients with chronic epididymitis. Chronic inflammation continues to activate dendritic cells and macrophages, producing cytokines that damage the epididymal epithelium, causing dendritic cells and macrophages to infiltrate the epididymal lumen, misrecognizing normal sperm, resulting in sperm capture and immune damage, which could be the possible mechanisms of phagocytosis of sperm cells under inflammatory.

A previous study identified a functional population of mononuclear phagocytes in the epididymal epithelium of mice responsible for maintaining the integrity of the blood-epididymis barrier (26), which has some similarities with our report: 1. It was observed that epididymal immune cells including macrophages and dendritic cells could clear sperms by morphological experiment; 2.There are a certain number of macrophages and dendritic cells in epididymal epithelium, and they have extensive signal communication with surrounding cells. On the other hand, our report is also very different from it: 1. Our research is human-based (chronic epididymitis patients), and theirs are mice-based; 2. Their study emphasizes the macrophages and dendritic cells clearance of damaged cells in normal conditions, while our study emphasizes the destruction of normal sperm by macrophages and dendritic cells in under the condition of abnormal activation of inflammation. 3. Their study emphasizes the role of immune cells and peripheral cells in epididymal epithelium of normal mice, while our study emphasizes abnormally activated immune cells penetrating the blood-epididymis barrier and directly causing immune damage to sperm in the lumen of epididymis.

Our report has some limitations. First, to confirm spermatozoa engulfment, electron microscope analysis would represent the gold standard, but our case was unable to fulfill it due to availability reasons. Our study is a retrospective case report study, we are unable to complement the electron microscope specimen, and the patients with chronic epididymitis undergoing epididymectomy are very rare in clinic, the specimen is invaluable. From this report, we observed that a large number of spermatozoa adhered to the cluster like structure in the epididymal lumen by hematoxylin-eosin staining, which indicated that spermatozoa might be captured by phagocytic immune cells. Immunofluorescence staining can be used to identify the types of immune cells that phagocytize the sperm. In addition, there is a big gap between the size of sperm and immune cells, which also helps us to distinguish them. Second, we could not ascertain inflammation to be the sole cause of oligoasthenospermia, because impairment of spermatogenesis by chronic inflammation, endocrine abnormalities, and genetic variation could also be the cause of oligoasthenospermia.

In summary, we described the first reported case that both dendritic cells and macrophages were found capturing spermatozoa in the lumen of cauda epididymis in an oligoasthenospermia man with chronic epididymitis. However, since impairment of spermatogenesis by chronic inflammation, endocrine abnormalities or genetic variation could not be ruled out, the precise process and molecular mechanisms of macrophage and dendritic cells associated spermatophagy in chronic epididymitis remains to be elucidated in future study.

## Data Availability Statement 

The original contributions presented in the study are included in the article/**Supplementary Material**. Further inquiries can be directed to the corresponding author.

## Ethics Statement 

The studies involving human participants were reviewed and approved by the Ethics Committee of the Union Hospital of Fujian Medical University. The patients/participants provided their written informed consent to participate in this study. Written informed consent was obtained from the individual for the publication of any potentially identifiable images or data included in this article.

## Author Contributions

ML drafted and edited the manuscript, and critically discussed the study. WZ drafted and edited the manuscript, and evaluated the immunological data. SZ drafted and edited the manuscript. SJ and ZL performed the hematoxylin-eosin staining and immunofluorescence analyses. XC prepared the epididymal tissue paraffin specimen. All authors contributed to the article and approved the submitted version.

## Funding

This study was supported by the Startup Fund for scientific research, Fujian Medical University (Grant number: 2016QH032); Fujian Natural Sciences Foundation (Grant number: 2017J01203); Joint Funds for the innovation of science and Technology, Fujian province (Grant number: 2017Y9023); Startup Fund for scientific research, Fujian Medical University (Grant number: 2018QH1044); Startup Fund for scientific research of bring in talents, Fujian Medical University (Grant number: 2020XH004).

## Conflict of Interest

The authors declare that the research was conducted in the absence of any commercial or financial relationships that could be construed as a potential conflict of interest.

## Supplementary Material

Supplementary Figure 1 Immune cells infiltrate in the interstitial of the epididymis.

## References

[1] Makar RS Toth TL. The evaluation of infertility. Am J Clin Pathol (2002) 117 Suppl:S95–103. 10.1309/W8LJ-K377-DHRA-CP0B 14569805

[2] Hamada A Esteves SC Nizza M Agarwal A. Unexplained male infertility: diagnosis and management. Int Braz J Urol (2012) 38:576–94. 10.1590/s1677-55382012000500002 23131516

[3] Poongothai J Gopenath TS Manonayaki S. Genetics of human male infertility. Singapore Med J (2009) 50:336–47. 19421675

[4] La Vignera S Vicari E Condorelli RA D’Agata R Calogero AE. Male accessory gland infection and sperm parameters (review). Int J Androl (2011) 34:e330–47. 10.1111/j.1365-2605.2011.01200.x 21696400

[5] Colpi GM Francavilla S Haidl G Link K Behre HM Goulis DG. European Academy of Andrology guideline Management of oligo-astheno-teratozoospermia. Andrology-US (2018) 6:513–24. 10.1111/andr.12502 30134082

[6] Schutte B El HN Kuhtz J Nanda I Gromoll J Hahn T. Broad DNA methylation changes of spermatogenesis, inflammation and immune response-related genes in a subgroup of sperm samples for assisted reproduction. A-US (2013) 1:822–9. 10.1111/j.2047-2927.2013.00122.x PMC4033565 23996961

[7] Schuppe HC Pilatz A Hossain H Diemer T Wagenlehner F Weidner W. Urogenital Infection as a Risk Factor for Male Infertility. Dtsch Arztebl Int (2017) 114:339–46. 10.3238/arztebl.2017.0339 PMC5470348 28597829

[8] Michel V Pilatz A Hedger MP Meinhardt A. Epididymitis: revelations at the convergence of clinical and basic sciences. Asian J Androl (2015) 17:756–63. 10.4103/1008-682X.155770 PMC4577585 26112484

[9] Sullivan R Mieusset R. The human epididymis: its function in sperm maturation. Hum Reprod Upd (2016) 22:574–87. 10.1093/humupd/dmw015 27307387

[10] Da SN Barton CR. Macrophages and dendritic cells in the post-testicular environment. Cell Tissue Res (2016) 363:97–104. 10.1007/s00441-015-2270-0 26337514 PMC4703462

[11] Cooper TG Yeung CH Jones R Orgebin-Crist MC Robaire B. Rebuttal of a role for the epididymis in sperm quality control by phagocytosis of defective sperm. J Cell Sci (2002) 115:5–7. 11801718 10.1242/jcs.115.1.5

[12] Jones R. Sperm survival versus degradation in the Mammalian epididymis: a hypothesis. Biol Reprod (2004) 71:1405–11. 10.1095/biolreprod.104.031252 15215193

[13] Holstein AF. Spermatophagy in the seminiferous tubules and excurrent ducts of the testis in Rhesus monkey and in man. ANDROLOGIA (1978) 10:331–52. 10.1111/j.1439-0272.1978.tb03044.x 102218

[14] Duan YG Zhang Q Liu Y Mou L Li G Gui Y. Dendritic cells in semen of infertile men: association with sperm quality and inflammatory status of the epididymis. Fertil Steril (2014) 101:70–77.e3. 10.1016/j.fertnstert.2013.09.006 24083871

[15] Duan YG Wang P Zheng W Zhang Q Huang W Jin F. Characterisation of dendritic cell subsets in chronically inflamed human epididymis. Andrologia (2016) 48:431–40. 10.1111/and.12463 26257153

[16] Kampf C Olsson I Ryberg U Sjostedt E Ponten F. Production of tissue microarrays, immunohistochemistry staining and digitalization within the human protein atlas. J Vis Exp (2012) 63:3620. 10.3791/3620 PMC3468196 22688270

[17] Horan AH Bedford JM. Development of the fertilizing ability of spermatozoa in the epididymis of the Syrian hamster. J Reprod Fertil (1972) 30:417–23. 10.1530/jrf.0.0300417 5073383

[18] Hedger MP. Immunophysiology and pathology of inflammation in the testis and epididymis. J Androl (2011) 32:625–40. 10.2164/jandrol.111.012989 PMC7166903 21764900

[19] Hedger MP Winnall WR. Regulation of activin and inhibin in the adult testis and the evidence for functional roles in spermatogenesis and immunoregulation. Mol Cell Endocrinol (2012) 359:30–42. 10.1016/j.mce.2011.09.031 21964464

[20] Hedger MP. Immunophysiology and pathology of inflammation in the testis and epididymis. J Androl (2011) 32:625–40. 10.2164/jandrol.111.012989 PMC7166903 21764900

[21] Fijak M Pilatz A Hedger MP Nicolas N Bhushan S Michel V. Infectious, inflammatory and ‘autoimmune’ male factor infertility: how do rodent models inform clinical practice? Hum Reprod Upd (2018) 24:416–41. 10.1093/humupd/dmy009 PMC6016649 29648649

[22] Lotti F Baldi E Corona G Lombardo F Maseroli E Degl’Innocenti S. Epididymal more than testicular abnormalities are associated with the occurrence of antisperm antibodies as evaluated by the MAR test. Hum Reprod (2018) 33:1417–29. 10.1093/humrep/dey235 29982596

[23] Munn DH Mellor AL. Indoleamine 2,3-dioxygenase and tumor-induced tolerance. J Clin Invest (2007) 117:1147–54. 10.1172/JCI31178 PMC1857253 17476344

[24] Da Silva N Cortez-Retamozo V Reinecker HC Wildgruber M Hill E Brown D. A dense network of dendritic cells populates the murine epididymis. Reproduction (2011) 141:653–63. 10.1530/REP-10-0493 PMC3657760 21310816

[25] Guiton R Henry-Berger J Drevet JR. The immunobiology of the mammalian epididymis: the black box is now open! Basic Clin Androl (2013) 23:8. 10.1186/2051-4190-23-8 25780570 PMC4349724

[26] Smith TB Cortez-Retamozo V Grigoryeva LS Hill E Pittet MJ Da SN. Mononuclear phagocytes rapidly clear apoptotic epithelial cells in the proximal epididymis. Andrology-US (2014) 2:755–62. 10.1111/j.2047-2927.2014.00251.x PMC4396827 25082073

