# Supplementary figures and images for: Case Report: Dendritic Cells and Macrophages Capture Sperm in Chronically Inflamed Human Epididymis
Source: Front Immunol. 2021 Feb 23;12:629680. doi: 10.3389/fimmu.2021.629680 (PMC7942197; doi:10.3389/fimmu.2021.629680)

## Supplementary Figure 1

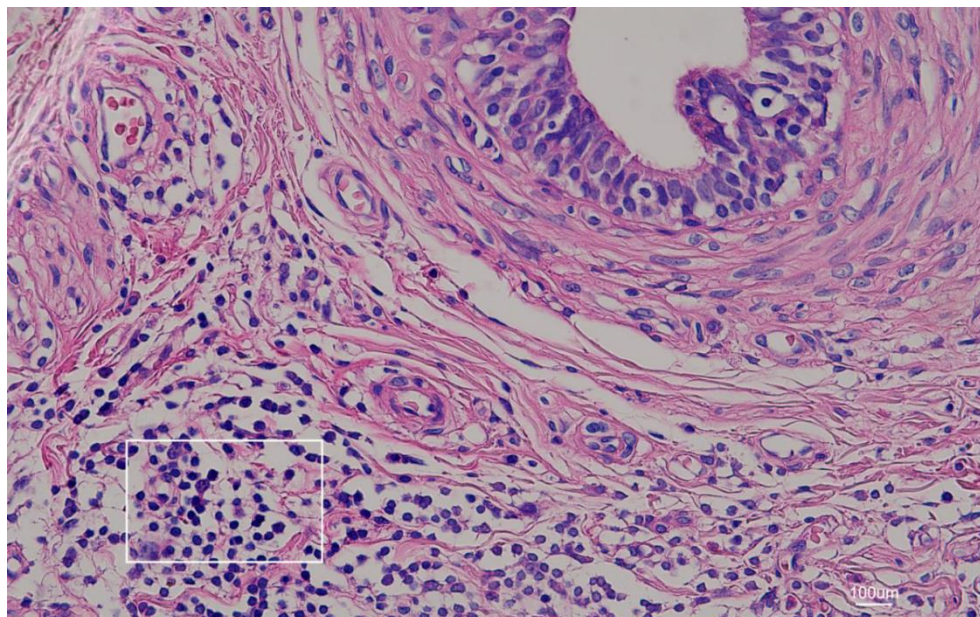

Supplement: Supplementary Figure 1 — Immune cells infiltrate in the interstitial of the epididymis. [file Image_1.pdf]
